# Comparing the expression levels of tripartite motif containing 28 in mild and severe COVID-19 infection

**DOI:** 10.1186/s12985-022-01885-0

**Published:** 2022-10-03

**Authors:** Rezvan Tavakoli, Pooneh Rahimi, Mojtaba Hamidi-Fard, Sana Eybpoosh, Delaram Doroud, Iraj Ahmadi, Enayat Anvari, Mohammadreza Aghasadeghi, Abolfazl Fateh

**Affiliations:** 1 Hepatitis and AIDS Department, Pasteur Institute of Iran, Tehran, Iran; 2 Viral Vaccine Research Center, Pasteur Institute of Iran, Tehran, Iran; 3 Department of Epidemiology and Biostatistics, Research Centre for Emerging and Reemerging Infectious Diseases, Pasteur Institute of Iran, Tehran, Iran; 4 Quality Control Department, Production and Research Complex, Pasteur institute of Iran, Tehran, Iran; 5 Department of Physiology, School of Medicine, Ilam University of Medical Science, Ilam, Iran; 6 Department of Mycobacteriology and Pulmonary Research, Pasteur Institute of Iran, Tehran, Iran; 7 Microbiology Research Center (MRC), Pasteur Institute of Iran, Tehran, Iran

**Keywords:** Tripartite motif containing 28, Coronavirus disease 2019, Mild infection, Severe infection

## Abstract

**Background:**

Tripartite motif-containing 28 (TRIM28) is an impressive regulator of the epigenetic control of the antiviral immune response. This study evaluated if the differential expression of TRIM28 correlates with the severity of coronavirus disease 2019 (COVID-19) infection.

**Methods:**

A total of 330 COVID-19 patients, including 188 mild and 142 severe infections, and 160 healthy controls were enrolled in this study. Quantitative real-time polymerase chain reaction (qPCR) was used to determine the expression levels of *TRIM28* in the studied patients.

**Results:**

TRIM28 mRNA levels were significantly lower in both groups of patients versus the control group and in the severe group indicated further reduction in comparison to mild infection. The multivariate logistic regression analysis showed the mean age, lower levels of low-density lipoprotein (LDL), high-density lipoprotein (HDL), cholesterol, lower 25-hydroxyvitamin D, and PCR cycle threshold (Ct) value and higher levels of erythrocyte sedimentation rate (ESR) and differential expression of TRIM28 were linked to the severity of COVID-19 infection.

**Conclusion:**

The results of this study proved that the downregulation of TRIM28 might be associated with the severity of COVID-19 infection. Further studies are required to determine the association between the COVID-19 infection severity and TRIM family proteins.

## Background

Coronavirus disease 2019 (COVID-19) caused by severe acute respiratory syndrome coronavirus 2 (SARS-CoV-2) is accompanied by different disease patterns from asymptomatic or mild to severe symptoms that can lead to mortality [1]. In addition to the advanced age or special health conditions, such as chronic lung disease, diabetes, or cardiovascular disease in patients, the host response to SARS-CoV-2 infection can inflict these different clinical manifestations [2]. The host response mechanisms in this disease are poorly understood, and there is a vital requirement to understand how the host’s immune system responds to this mysterious virus [3].

The innate immune response to the virus is triggered by recognizing viral RNA and glycoproteins by pathogen recognition receptors (PRRs) and toll like receptors (TLRs) that activate signal transduction cascade to subsequently express interferons (IFNs) [4, 5]. This results in the nuclear translocation of the signal transducers and activators of transcription (STATs) and upregulation expression of numerous IFN-stimulated genes (ISGs), such as proinflammatory cytokines [6]. However, the principal mechanisms of this overexpression of IFNs and cytokines during SARS-CoV-2 infection are still not entirely understood [7].

Previous studies have shed light on tripartite motif-containing 28 (TRIM28) as impressive regulators of the epigenetic control of the antiviral immune response [8–10]. The TRIM28 is a negative immune regulator moderating IFNs and cytokine expression during infection with highly pathogenic avian influenza virus (HPAIV). It was shown that unrestrained inflammatory responses are more likely the reason for severe infection with HPAIV, which can lead to mortality [10].

The TRIM28 belongs to the subfamily VI of TRIM family proteins with ubiquitin E3 ligases activity in their N-terminal, and three unique functional domains in their C-terminal that put TRIM28 as a transcriptional regulator in this subfamily with TRIM24, TRIM33 and TRIM66. Heterochromatin protein 1 binding domain (HP1 BD) in C-terminal of TRIM28 can change the epigenetic state as a transcriptional repressor and affect the silencing of genes and retroelements. This repressing function of TRIM28 plays an important role on silencing viral transcription and replication.[11]. The TRIM proteins have a pivotal role in antiviral defense directly by restricting the replication of the virus and/or indirectly through modulating antiviral cytokine responses [12]. They are conjugated to the lysine residues of a target protein and modify them by phosphorylation, ubiquitination, and proteasome-driven degradation. Although most TRIM proteins activate as an immune enhancer, TRIM28 was reported as an immune suppressor which downregulates the activity of multiple immune-related transcription factors, including interferon regulatory factor 7 (IRF7), IRF5 and IRF1 [13]. It was demonstrated that *TRIM28* can restrict the replication of human immunodeficiency virus type 1 (HIV-1) by inhibiting its integrase protein and repressing the HIV-1 L promoter. Additionally, *TRIM28* can induce latency of herpesvirus, Epstein-Barr virus, and endogenous retrovirus. Recently, it has been shown that *TRIM28* has co-expression with SARS-CoV-2 receptors and can impress cell entry by affecting interferon-γ (IFN-γ)-induced angiotensin-converting enzyme 2 (*ACE2*) gene expression [14, 15].

The TRIM28, also called KRAB-associated protein-1 (KAP1) or transcriptional intermediary factor 1β (TIF1-β), is a nuclear corepressor of Kruppel-associated box zinc-finger proteins (KRAB-ZFPs) that impresses STAT1 for ubiquitination as a posttranslational modification of viral and host protein [16].

The effect of the *TRIM28* gene in COVID-19 as a new disease seems to be interesting; therefore, this study aimed to evaluate the expression of the *TRIM28* gene in the blood samples of patients with mild and severe COVID-19 infections.

## Materials and methods

### Study population

The study population included 330 blood samples from patients with COVID-19 infection, including mild infection (the patients indicating light clinical symptoms accompanied by fever and no evidence of pneumonia or mild respiratory symptoms on computed tomography examination) and severe infection (the patients with shortness of breath, oxygen saturation less than 92%, shock, acute respiratory failure requiring mechanical ventilation machine, and need to be admitted to an intensive care unit). Furthermore, 160 blood samples of healthy individuals as a control group were collected at Pasteur Institute of Iran.

The patients’ samples were collected within March 2020 to September 2020, and the samples of healthy individuals belonged to before the COVID-19 pandemic. The patients did not have any underlying medical conditions, including heart diseases, diabetes, cancer, chronic respiratory diseases, and allergic diseases.

The laboratory parameters, including real-time PCR Ct values, alanine aminotransferase (ALT), aspartate aminotransferase (AST), alkaline phosphatase (ALP), blood urea nitrogen (BUN), serum creatinine, uric acid, triiodothyronine (T3), thyroxine (T4), thyroid-stimulating hormone (TSH), platelets, 25-hydroxyvitamin D, C-reactive protein (CRP), white blood cells (WBC), hemoglobin, erythrocyte sedimentation rate (ESR), fasting blood glucose (FBS), low-density lipoprotein (LDL), cholesterol, triglyceride (TG), and high density lipoprotein (HDL) were extracted from the patients’ records.

**Evaluation of the Expression of*TRIM28*mRNA by Real-Time PCR**.

Total RNA was extracted using Trizol Reagent (QIAGEN, USA) according to the manufacturer’s instructions and quantified by a NanoDrop spectrophotometer. The complementary DNA (cDNA) was synthesized according to the manufacture of the kit (Yekta Tajhiz, Iran) by (1 µg) of total RNA treated by the DNase I enzyme (Thermofisher, USA). The cDNA was amplified using a real-time polymerase chain reaction (PCR) machine (Corbett Rotor-Gene 6000, QIAGEN, USA) with SYBR Green qPCR Master Mix (Ampliqon, Denmark) and hypoxanthine phosphoribosyltransferase 1 (HPRT1) gene as a reference. The primer sequences were designed as an exon junction or separate exon by Beacon designer software (Version 8 Primer, Biosoft, USA). The primer sequences for the TRIM28 gene were: 5’-GGACCACCAGTACCAGTTC-3’ (forward), and 5’-CCATCTTGACATCCACTTGC-3’ (reverse) and for *HPRT1* gene was: 5’-TGCTGAGGATTTGGAAAGGG-3’ (forward), and 5’-ACAGAGGGCTACAATGTGATG-3’ (reverse).

### Statistical analysis

Processing real-time PCR data was performed by the ΔΔCt method. Statistical analysis was applied via the GraphPad Prism statistical software (version 9.3.1) and SPSS for Windows version (version 22.0.) (SPSS, Inc., Chicago, IL, USA). Nonparametric data are expressed as medians and interquartile ranges (IQR). The Shapiro-Wilk test was used to evaluate the normality of ordinal variables. Pearson’s Chi-square test and Mann-Whitney U test were also used to assess quantitative and continuous variables, respectively. A multivariate logistic regression analysis was carried out by the Hosmer-Lemeshow test to assess the association between COVID-19 resistance and several risk factors for susceptibility. The area under the receiver-operating characteristic curve (AUC-ROC) analysis was applied to evaluate the impact of *TRIM28* gene expression on resistance and susceptibility to COVID-19. A *p*-value less than 0.05 was considered statistically significant.

## Results

### Baseline characteristics of COVID-19 patients

Table 1 shows the patients’ laboratory and clinical characteristics. The studied subjects were divided into three groups, including mild patients (n = 188), severe patients (n = 142), and healthy controls (n = 160). The mean age values of mild patients, severe patients, and healthy controls were 53.7 ± 11.7, 61.2 ± 2.8, and 54.2 ± 12.1 years, respectively. Overall, 174 and 83 patients in COVID-19 patients and healthy controls were male, respectively. Severe COVID-19, compared to mild infection, was significantly correlated with high levels of CRP (*P* < 0.001) and ESR (*P* < 0.001) and lower levels of 25-hydroxyvitamin D (*P* < 0.001), LDL (*P* < 0.001), TG *(P* < 0.001), cholesterol (*P* < 0.001), and low real-time PCR Ct value (*P* = 0.018).

**Table 1:** Comparison laboratory parameters between mild and severe patients infected with COVID-19

Variables	Mild patients (n = 188)	Severe patients (n = 142)	Control group (n = 160)	*P*-value
Mean age ± SD	53.7 ± 11.7	61.2 ± 12.8	54.2 ± 12.1	0.219
Gender (male/female)	108/80 (57.4/42.6%)	66/76 (46.5/53.5%)	82/78 (51.2/48.8%)	0.187
ALT, IU/L (mean ± SD) (Reference range: 5–40)	29.7 ± 19.1	32.5 ± 20.3	29.5 ± 20.4	0.761
AST, IU/L (mean ± SD) (Reference range: 5–40)	31.1 ± 16.9	34.6 ± 19.7	28.6 ± 16.7	0.367
ALP, IU/L (mean ± SD) (Reference range: up to 306)	180.4 ± 98.9	172.8 ± 82.6	212.8 ± 99.6	0.901
Cholesterol, mg/dL (mean ± SD) (Reference range: 50–200)	171.9 ± 53.9	109.1 ± 29.4	167.9 ± 49.8	< 0.001*
TG, mg/dL (mean ± SD) (Reference range: 60–165)	173.1 ± 62.4	125.9 ± 63.4	162.1 ± 58.4	< 0.001*
LDL, mg/dL (mean ± SD) (Reference range: up to 150)	125.1 ± 49.1	56.4 ± 19.3	110.5 ± 28.7	< 0.001*
HDL, mg/dL (mean ± SD) (Reference range: >40)	31.0 ± 12.1	33.1 ± 12.9	39.1 ± 13.1	0.287
WBC, 10^9^/L (mean ± SD) (Reference range: 4000–10,000)	7443.6 ± 2524.5	7823.1 ± 2906.9	6842.1 ± 2102.1	0.882
CRP, mg/L (mean ± SD) (Reference range: <10 mg/L Negative)	58.8 ± 21.5	74.3 ± 14.9	9.3 ± 2.9	< 0.001*
ESR, mm/1st h (mean ± SD) (Reference range: 0–15)	51.0 ± 17.4	61.5 ± 12.4	11.5 ± 4.7	< 0.001*
FBS, mg/dL (mean ± SD) (Reference range: 70–100)	103.5 ± 40.0	102.5 ± 30.1	99.5 ± 28.2	0.751
Platelets × 1000/cumm (mean ± SD) (Reference range: 140,000–400,000)	179 ± 67	188 ± 84	185 ± 81	0.689
T3, ng/dL (mean ± SD) (Reference range: 2.3–4.2)	2.4 ± 1.3	1.7 ± 0.9	2.1 ± 1.1	0.752
T4, mcg/dL (mean ± SD) (Reference range: 5.6–13.7)	8.6 ± 7.1	9.9 ± 7.2	10.1 ± 7.9	0.284
TSH, mu/L (mean ± SD) (Reference range: 0.4–4.5)	3.1 ± 1.7	3.3 ± 1.8	2.8 ± 1.1	0.456
Hemoglobin, g/dL (mean ± SD) (Reference range: 12–18)	13.1 ± 2.5	12.2 ± 1.7	14.2 ± 2.7	0.417
BUN, mg/dL (mean ± SD) (Reference range: 15–45)	38.1 ± 7.1	36.9 ± 6.9	32.1 ± 5.9	0.086
Creatinine, mg/dL (mean ± SD) (Reference range: 0.6–1.4)	1.1 ± 0.6	1.0 ± 0.4	1.2 ± 0.7	0.258
25-hydroxy vitamin D, ng/mL (mean ± SD) (Sufficiency: 21–150)	34.7 ± 13.8	19.1 ± 9.7	31.2 ± 11.7	< 0.001*
Real-time PCR Ct values	28.8 ± 8.9	12.8 ± 7.4	-	0.018*

ALT, alanine aminotransferase; AST, aspartate aminotransferase; ALP, alkaline phosphatase; TG, triglyceride; LDL, low density lipoprotein; HDL, high density lipoprotein; WBC, white blood cells; CRP, C-reactive protein; ESR, erythrocyte sedimentation rate; FBS, fasting blood glucose; T3, triiodothyronine; T4, thyroxine; TSH, Thyroid-stimulating hormone; BUN, Blood urea nitrogen; Ct, cycle threshold; SD, standard deviation. *Statistically significant (< 0.05)

**Relationship between*TRIM28*gene expression and COVID-19 infection severity**.

**Fig. 1:**
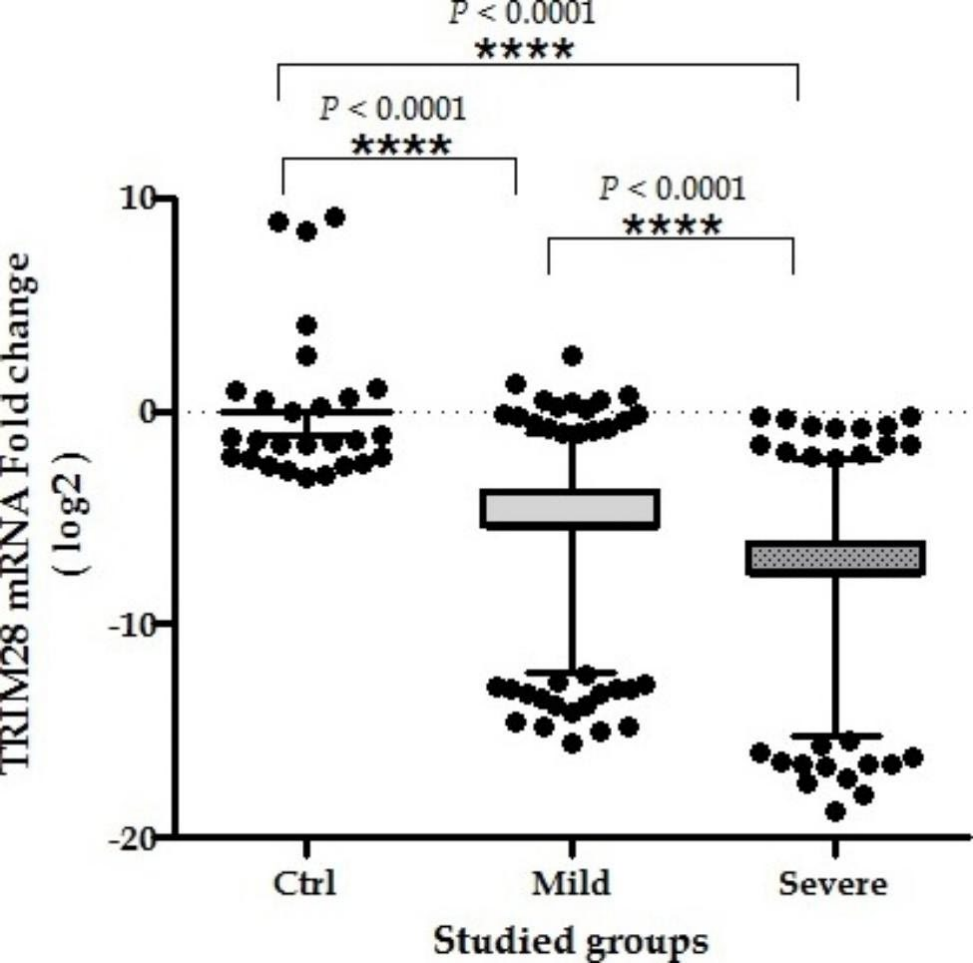
Expression of *TRIM28* in 330 whole blood samples from 3 groups: Mild and Severe COVID-19 patients and 160 uninfected individuals as control group. Ctrl: control group Mild: infected patients with mild symptoms. Severe: infected patients with severe disease. A horizontal line shows the median values with SEM error bars. Median values and interquartile range (IQR) for *TRIM28* Ctrl: median 0.00, IQR − 3.12, 9.85; Mild: median − 5.47, IQR − 15.61, 2.59; Severe: median: -7.64, IQR − 18.79, -0.3. Statistical analysis: the Mann-Whitney test was used to compare the transcriptional levels of each group of patients with control group

**Fig. 2:**
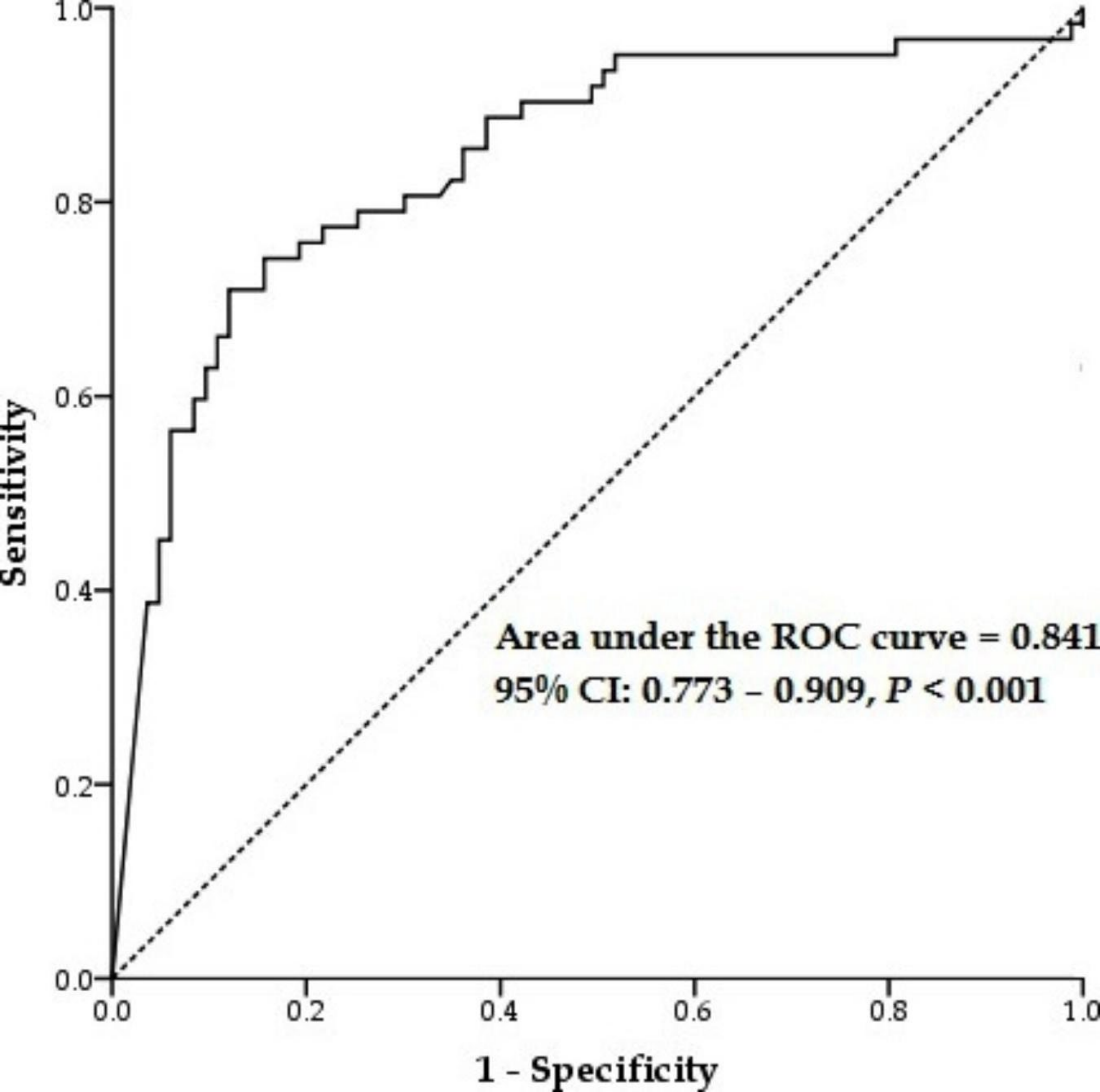
ROC curve with the *TRIM28* gene expression for prediction the severity of COVID-19 infection

The expression of the *TRIM28* mRNA gene in both groups of patients showed a significant decline in comparison to that of the control group. The group with severe symptoms of COVID-19 containing hospitalized patients demonstrated more reduction in comparison to the mild infection group, including outpatient individuals (Fig. 1). Moreover, the AUC-ROC value for *TRIM28* gene expression was 0.841, suggesting that the expression of this gene is commonly crucial for viral infection resolution (Fig. 2).

## Factors related to COVID-19 infection severity

The multivariate logistic regression analysis evaluated the factors associated with COVID-19 infection severity. The severity of COVID-19 infection was associated with mean age (OR 0.911, 95% CI 0.875–0.998, *P* = 0.021), LDL (OR 0.917, 95% CI 0.856–0.981, *P* = 0.012), cholesterol (OR 0.919, 95% CI 0.845–0.998, *P* = 0.046), HDL (OR 1.283, 95% CI 1.070–1.539, *P* = 0.007), 25-hydroxyvitamin D (OR 1.180, 95% CI 1.106–1.912, *P* = 0.018), ESR (OR 0.801, 95% CI 0.679–0.945, *P* = 0.009), real-time PCR Ct values (OR 2.181, 95% CI 1.129–2.925, *P* = 0.033), and *TRIM28* gene expression (OR 0.6456, 95% CI 0.457–0.911, *P* = 0.013) (Table 2).

**Table 2:** Factors associated with severe patients infected with COVID-19

Factors		
**Baseline Predictors**	**OR (95% CI)**	***P*** **-value**
Mean age ± SD	0.911 (0.875–0.998)	0.021*
LDL (mg/dL)	0.917 (0.856–0.981)	0.012*
Cholesterol, mg/dL	0.919 (0.845–0.998)	0.046*
HDL (mg/dL)	1.283 (1.070–1.539)	0.007*
25-hydroxyvitamin D, (ng/Ml)	1.180 (1.106–1.912)	0.018*
ESR, (mm/1st h)	0.801 (0.679–0.945)	0.009*
Real-time PCR Ct values	2.181 (1.129–2.925)	0.033*
*TRIM28* gene expression	0.645 (0.457–0.911)	0.013*

LDL, low density lipoprotein; HDL, high density lipoprotein; ESR, erythrocyte sedimentation rate; Ct, cycle threshold; *TRIM28*, tripartite Motif Containing 28; SD, standard deviation; *Statistically significant (< 0.05)

## Discussion

The present study evaluated the relationship between *TRIM28* gene expression and the progression of COVID-19 infection. Our results showed a meaningful reduction of TRIM28 in patients with COVID-19 in both studied groups and proposed a possible relationship between *TRIM28* expression and the rate of COVID-19 severity. Growing data indicate that *TRIM28* showed a reduction in COVID-19 disease [17–19]. Moreover, the co-culture of lung epithelial cells and NK cells showed that *TRIM28* can be inhibited by NK cell-derived factors and causes the induction of *ACE2* expression in lung epithelial cells [19]. In addition, Yinfang Wang et al. reported that in vitro TRIM28 knockdown enhanced interferon gamma-induced *ACE2* expression via upregulating interferon-gamma receptor 2 (IFNGR2) in both A549 and primary alveolar epithelial cells. Therefore, the promotion of *ACE2* expression due to NK cell function and decrease in *TRIM28* raise the possibility of infection with SARS-CoV-2 in lung epithelial cells and intensify lung inflammation and infection [19]. It seems that the low expression of *TRIM28* causes an increase in the ACE2 expression and SARS-CoV-2 entry in the cells. As a result, the decrease in the expression of TRIM28 in both studied groups compared to the control group may be due to the increase in the expression of *ACE2* and the entry of the virus into the cell.

The *TRIM28* gene significantly decreased in children with severe COVID-19 in comparison to children with mild infection in the blood samples [17]. The RNA-sequencing analysis of transcriptional response to SARS-CoV-2 in cells, animal models, and lung tissue clinical samples of COVID-19 patients also showed a reduction in *TRIM28* gene [18].

Recently, Blanco-Melo D et al. characterized immune cells by analyzing a single-cell RNA sequencing dataset from bronchoalveolar fluid in patients with severe COVID-19 and revealed that different types of immune cells were identified in patients [18]. Among various identified cells, natural killer (NK) cells lead to a balanced immune response by removing infected cells quenching dendritic cell and T cell activity due to SARS-CoV-2 infection [20]. Krischuns and et al. reported that inactive TRIM28 due to phosphorylation of TRIM28 at serine 473 in a signaling cascade leads to an intensification of persistent immune response by boosting IFN-β, IL-6 and IL-8 expression which may result to extreme immune cells activity and cytokine-mediated tissue damage during HPAIV infection [18]. On the other side, it showed *TRIM28* co-expression with *ACE2* and transmembrane serine protease 2 (*TMPRSS2*) in type II pneumocystis. Since binding the spike glycoprotein of Coronavirus to their cellular receptors and then fusion between viral and host cell membranes is the first step of viral infection, increasing the expression of these receptors can impress on the rate of viral infection [7]. Ubiquitination alterations in both viral and host proteins were observed in SARS-CoV-2-infected cells and the degradation of *ACE2* receptor by E3 ligase ubiquitination was improved [7]. As a regards TRIM28 is an E3 ligase, and had higher expression in patients with mild symptoms compared to severe cases, it may contribute to the downregulation of the major SARS-CoV-2 entry receptor and decreasing the severity of infection in mild group.

On the other hand, it was shown that *TRIM28* could restrictHIV-1 replication by inhibiting the activation of HIV-1 integrase [21]. Moreover, *TRIM28* was reported to inhibit Moloney murine leukemia virus (M-MLV) in pluripotent cells as an integral component of the silencing complex by bounding to the primer binding site of M-MLV and repressing transcription from the viral promoter [22].

The current study demonstrated a significant reduction in patients with severe infection compared to patients with mild infection and healthy control group. More *TRIM28* expression in mildly symptomatic individuals may suppress inflammatory genes in this group, while its more defective activation in severe group causes the high inflammation in cells. It seems that TRIM28 more likely has an important role in the control of high inflammatory response and might inhibit the replication of SARS-CoV-2 by the restriction of *ACE2* receptor in lung epithelial cells.

The results of this study indicated that the severity of COVID-19 infection was significantly correlated with the low levels of lipid profiles, including LDL, HDL, and cholesterol, and real-time PCR cycle threshold (Ct) value. A meta-analysis study demonstrated significantly decreased levels of LDL, HDL, and total cholesterol in the severe patients, compared to those of nonsevere patients in a random effect model (REM). Additionally, REM results indicated significantly lower levels of lipid profiles in the dead patients, compared to those of the survivors [23]. However, the main mechanism responsible for the decrease in lipid profile levels in severe COVID-19 patients is not clearly understood. There are several hypotheses in this regard. Firstly, the liver has a vital role in lipid metabolism, and SARS-CoV-2 might damage the liver, thereby impairing the absorption and biosynthesis of lipoproteins [24]. Secondly, an important feature of COVID-19 patients is hyperinflammation, especially in patients with severe infection or dead patients, which alters lipid metabolism. Interleukin (IL)-6, IL-1β, and tumor necrosis factor-alpha as proinflammatory cytokines have been indicated to modulate lipid metabolism by altering the liver’s function and reducing cholesterol efflux and transport [14, 15]. Thirdly, the inflammatory response caused by the virus can also alter vascular permeability, leading to the leakage of cholesterol molecules into tissues, such as the alveolar spaces for secretion. Secretions contain high levels of protein and cholesterol [25].

There is now considerable evidence to suggest a significant correlation between the insufficiency and deficiency of 25-hydroxyvitamin D and COVID-19 severity [26]. 25-hydroxyvitamin D is correlated with the immunity of both B and T cells. In general, T cell responses play a key role in fighting viral infections. The evidence suggests that patients with severe COVID-19 are characterized by functional T cells exhaustion, and the improvement of vitamin D status can reduce this process via immunomodulation [27].

A low Ct of real-time PCR Ct values was observed in the present examined severs patients. Some reports have indicated that this parameter increases the hospitalization risk and mortality [28]. Furthermore, a study showed a significant correlation between real-time PCR Ct values and secondary transmission. It was suggested that by real-time PCR Ct values or viral load calculations can help modify decision-making, such as shorter isolation [29].

The limitations of this study were the relatively small sample size, lack of evaluation of *TRIM28* expression in patients’ tissues, and lack of evaluation of the amount of TRIM28 protein in the patient’s serum.

## Conclusion

The current study’s findings revealed a potential correlation between the downregulation of *TRIM28* gene expression with the severity of COVID-19 infection, the lower levels of 25-hydroxyvitamin D, lipid profiles, and real-time PCR Ct values. Further studies are recommended to confirm the aforementioned findings.

## Data Availability

All data generated or analyzed during this study are included in this published article.

## Abbreviations

TRIM28: Tripartite motif-containing 28.
COVID-19: coronavirus disease 2019.
PCR: polymerase chain reaction.
LDL: low-density lipoprotein.
HDL: high-density lipoprotein.
Ct: cycle threshold.
ESR: erythrocyte sedimentation rate.
SARS-CoV-2: Severe acute respiratory syndrome coronavirus-2.
PRRs: pathogen recognition receptors.
TLR: toll like receptors.
IFNs: interferons.
STATs: signal transducers and activators of transcription.
ISGs: IFN-stimulated genes.
HPAIV: highly pathogenic avian influenza virus.
IRF7: interferon regulatory factor 7.
KAP1: KRAB-associated protein-1.
TIF1-β: transcriptional intermediary factor 1β.
KRAB-ZFPs: Kruppel-associated box zinc-finger proteins.
ALT: alanine aminotransferase.
AST: aspartate aminotransferase.
ALP: alkaline phosphatase.
BUN: blood urea nitrogen.
T3: triiodothyronine.
T4: thyroxine.
TSH: thyroid-stimulating hormone.
CRP: C-reactive protein.
WBC: white blood cells.
FBS: fasting blood glucose.
TG: triglyceride.
cDNA: complementary DNA.
HPRT1: hypoxanthine phosphoribosyltransferase 1.
IQR: interquartile range.
AUC-ROC: area under the receiver-operating characteristic curve.
NK: natural killer.
ACE2: angiotensin-converting enzyme 2.
TMPRSS2: transmembrane serine protease 2.
HIV-1: human immunodeficiency virus type 1.
M-MLV: Moloney murine leukemia virus.
REM: random effect model.

## References

[1] Tsabouri S, Makis A, Kosmeri C, Siomou E (2021). Risk factors for severity in children with coronavirus disease 2019: a comprehensive literature review. Pediatr Clin.

[2] Chow N, Fleming-Dutra K, Gierke R, Hall A, Hughes M, Pilishvili T (2020). CDC COVID-19 Response Team. Preliminary estimates of the prevalence of selected underlying health conditions among patients with coronavirus disease 2019—United States, February 12–March 28, 2020. MMWR Morb Mortal Wkly Rep.

[3] Chen F, Zhang Y, Sucgang R, Ramani S, Corry D, Kheradmand F, Creighton CJ (2021). Meta-analysis of host transcriptional responses to SARS-CoV-2 infection reveals their manifestation in human tumors. Sci Rep.

[4] Alexopoulou L, Holt AC, Medzhitov R, Flavell RA (2001). Recognition of double-stranded RNA and activation of NF-κB by Toll-like receptor 3. Nature.

[5] Pichlmair A, e Sousa CR (2007). Innate recognition of viruses. Immunity.

[6] Tisoncik JR, Korth MJ, Simmons CP, Farrar J, Martin TR, Katze MG (2012). Into the eye of the cytokine storm. Microbiol Mol Biol Rev.

[7] Liao M, Liu Y, Yuan J, Wen Y, Xu G, Zhao J, Cheng L, Li J, Wang X, Wang F (2020). Single-cell landscape of bronchoalveolar immune cells in patients with COVID-19. Nat Med.

[8] Kamitani S, Ohbayashi N, Ikeda O, Togi S, Muromoto R, Sekine Y, Ohta K, Ishiyama H, Matsuda T (2008). KAP1 regulates type I interferon/STAT1-mediated IRF-1 gene expression. Biochem Biophys Res Commun.

[9] Gehrmann U, Burbage M, Zueva E, Goudot C, Esnault C, Ye M, Carpier J-M, Burgdorf N, Hoyler T, Suarez G: Critical role for TRIM28 and HP1β/γ in the epigenetic control of T cell metabolic reprograming and effector differentiation. *Proceedings of the National Academy of Sciences* 2019, 116:25839–25849. 10.1073/pnas.1901639116 PMC6925996 31776254

[10] Krischuns T, Günl F, Henschel L, Binder M, Willemsen J, Schloer S, Rescher U, Gerlt V, Zimmer G, Nordhoff C. Phosphorylation of TRIM28 enhances the expression of IFN-β and proinflammatory cytokines during HPAIV infection of human lung epithelial cells. Frontiers in immunology 2018:2229. 10.3389/fimmu.2018.02229 PMC6172303 30323812

[11] Nisole S, Stoye JP, Saïb A (2005). TRIM family proteins: retroviral restriction and antiviral defence. Nat Rev Microbiol.

[12] Hatakeyama S (2017). TRIM family proteins: roles in autophagy, immunity, and carcinogenesis. Trends Biochem Sci.

[13] Liang Q, Deng H, Li X, Wu X, Tang Q, Chang T-H, Peng H, Rauscher FJ, Ozato K, Zhu F (2011). Tripartite motif-containing protein 28 is a small ubiquitin-related modifier E3 ligase and negative regulator of IFN regulatory factor 7. J Immunol.

[14] Ritchie AI, Singanayagam A (2020). Immunosuppression for hyperinflammation in COVID-19: a double-edged sword?. The Lancet.

[15] Zhang W, Zhao Y, Zhang F, Wang Q, Li T, Liu Z, Wang J, Qin Y, Zhang X, Yan X (2020). The use of anti-inflammatory drugs in the treatment of people with severe coronavirus disease 2019 (COVID-19): The Perspectives of clinical immunologists from China. Clin Immunol.

[16] Friedman JR, Fredericks WJ, Jensen DE, Speicher DW, Huang X-P, Neilson EG, Rauscher FJ (1996). KAP-1, a novel corepressor for the highly conserved KRAB repression domain. Genes Dev.

[17] Tovo P-A, Garazzino S, Daprà V, Pruccoli G, Calvi C, Mignone F, Alliaudi C, Denina M, Scolfaro C, Zoppo M (2021). COVID-19 in children: expressions of type I/II/III interferons, TRIM28, SETDB1, and endogenous retroviruses in mild and severe cases. Int J Mol Sci.

[18] Blanco-Melo D, Nilsson-Payant BE, Liu W-C, Uhl S, Hoagland D, Møller R, Jordan TX, Oishi K, Panis M, Sachs D (2020). Imbalanced Host Response to SARS-CoV-2 Drives Development of COVID-19. Cell.

[19] Wang Y, Fan Y, Huang Y, Du T, Liu Z, Huang D, Wang Y, Wang N, Zhang P (2021). TRIM28 regulates SARS-CoV-2 cell entry by targeting ACE2. Cell Signal.

[20] Wen W, Su W, Tang H, Le W, Zhang X, Zheng Y, Liu X, Xie L, Li J, Ye J (2020). Immune cell profiling of COVID-19 patients in the recovery stageby single-cell sequencing. Cell Discovery.

[21] Allouch A, Di Primio C, Alpi E, Lusic M, Arosio D, Giacca M, Cereseto A (2011). The TRIM family protein KAP1 inhibits HIV-1 integration. Cell Host Microbe.

[22] Lee A, CingÖz O, Sabo Y, Goff SP (2018). Characterization of interaction between Trim28 and YY1 in silencing proviral DNA of Moloney murine leukemia virus. Virology.

[23] Mahat RK, Rathore V, Singh N, Singh N, Singh SK, Shah RK, Garg C (2021). Lipid profile as an indicator of COVID-19 severity: a systematic review and meta-analysis. Clin Nutr ESPEN.

[24] Wei X, Zeng W, Su J, Wan H, Yu X, Cao X, Tan W, Wang H (2020). Hypolipidemia is associated with the severity of COVID-19. J Clin Lipidol.

[25] Heffner JE, Sahn SA, Brown LK (2002). Multilevel likelihood ratios for identifying exudative pleural effusions. Chest.

[26] Bae JH, Choe HJ, Holick MF, Lim S. Association of vitamin D status with COVID-19 and its severity. Reviews in Endocrine and Metabolic Disorders 2022:1–21. 10.1007/s11154-021-09705-6 PMC8724612 34982377

[27] Yang J, Zheng Y, Gou X, Pu K, Chen Z, Guo Q, Ji R, Wang H, Wang Y, Zhou Y (2020). Prevalence of comorbidities and its effects in patients infected with SARS-CoV-2: a systematic review and meta-analysis. Int J Infect Dis.

[28] Rahimi P, Tarharoudi R, Rahimpour A, Mosayebi Amroabadi J, Ahmadi I, Anvari E, Siadat SD, Aghasadeghi M, Fateh A (2021). The association between interferon lambda 3 and 4 gene single-nucleotide polymorphisms and the recovery of COVID-19 patients. Virol J.

[29] Al Bayat S, Mundodan J, Hasnain S, Sallam M, Khogali H, Ali D, Alateeg S, Osama M, Elberdiny A, Al-Romaihi H (2021). Can the cycle threshold (Ct) value of RT-PCR test for SARS CoV2 predict infectivity among close contacts?. J Infect Public Health.

