# Correction: comparing the expression levels of tripartite motif containing 28 in mild and severe COVID-19 infection

**DOI:** 10.1186/s12985-022-01903-1

**Published:** 2022-10-31

**Authors:** Rezvan Tavakoli, Pooneh Rahimi, Mojtaba Hamidi-Fard, Sana Eybpoosh, Delaram Doroud, Iraj Ahmadi, Enayat Anvari, Mohammadreza Aghasadeghi, Abolfazl Fateh

**Affiliations:** 1 Hepatitis and AIDS Department, Pasteur Institute of Iran, Tehran, Iran; 2 Viral Vaccine Research Center, Pasteur Institute of Iran, Tehran, Iran; 3 Department of Epidemiology and Biostatistics, Research Centre for Emerging and Reemerging Infectious Diseases, Pasteur Institute of Iran, Tehran, Iran; 4 Quality Control Department, Production and Research Complex, Pasteur institute of Iran, Tehran, Iran; 5 Department of Physiology, School of Medicine, Ilam University of Medical Science, Ilam, Iran; 6 Department of Mycobacteriology and Pulmonary Research, Pasteur Institute of Iran, Tehran, Iran; 7 Microbiology Research Center (MRC), Pasteur Institute of Iran, Tehran, Iran

**Correction: Virology Journal (2022) 19:156**

10.1186/s12985-022-01885-0.

Following publication of the original article [1], the authors identified an error in the author name of Sana Eybpoosh.

The incorrect author name is: Sana Eaybpoush.

The correct author name is: Sana Eybpoosh.

The original article has been corrected.
